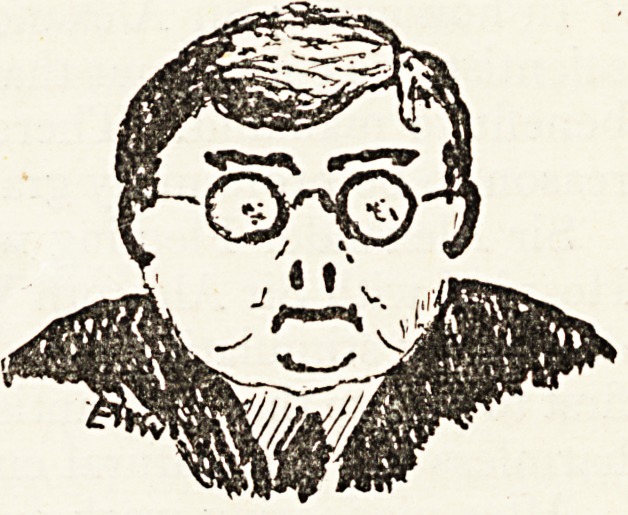# Meetings of Societies

**Published:** 1950-01

**Authors:** 


### Meetings of Societies

### Bristol Medico-Chirurgical Society

*Committee* 1949–50

*President:* Dr. H. J. Orr-Ewing. *President Elect:* Mr. W. A. Jackman. *Treasurer:* Dr. Sutton. *Secretary:* Mr. A. L. Eyre-Brook. *Editor of "Journal":* Mr. E. Watson-Williams. *Assistant Editor:* Dr. N. S. Craig (*ex officio*). *Library representatives:* Professors Milnes Walker, Perry, and Drew-Smythe.

*Elected members:* Dr. Gornall, Dr. Kersley, Dr. Tryon, Dr. Lennox Robertson, Dr. Rowley, Dr. Marwood, Dr. Lucas, Dr. Silvey.

The Annual General Meeting was held in the University on Wednesday, October 12th, 1949, when the retiring President installed his successor Dr. H. J. Orr Ewing. Dr. Orr Ewing's Presidential address appears on page 1 of this issue. The officers of the society presented their Annual Reports which were received. Details included in the Secretary's Report have appeared in our pages from time to time. There are now 382 members. The Honorary Treasurer's Report is summarized in the balance sheets which we hope to publish shortly. For the first time for many years vacancies in the Committee were filled by ballot as is laid down in the Laws.

*Editor's Report*

During the past year we have secured an increased number of non-member subscribers. The *Journal* has handed over to the University Library eighty-five "Exchange" publications priced at £105 (before devaluation) and the books reviewed, at £37 11s. 6d. This sum of £142 11s. 6d. represents "invisible earnings" by the *Journal* for the Society.

We have now been paid the arrears of subscriptions for the years 1939–45, £100: and the sum of £170 voted in 1947 to cover the deficit which arose from the great increase in the cost of printing. This increase has continued and although the accounts for the year have not yet been audited I may tell you that the nett cost of production is a little over £100 for each quarterly issue. Fortunately we have covered half this by receipts from advertisements, which are now about three times as great as in 1947. We expect therefore to show a modest profit on the year's work and can look forward to a partial relaxation of the severe limitations that had recently to be imposed—in fact, to a larger and we hope a better *Journal*.

The Second Meeting of the Society on Wednesday, November 9th, was devoted to a Clinical Meeting at the Bristol General Hospital.

Cases were shown by: Mr. A. Wilfred Adams, Mr. J. Angell James, Dr. C. D. Evans, Mr. K. H. Pridie, Mr. Ashton Miller, Professor A. V. Neale, Dr. Beryl Corner, Dr. A. E. W. McLachlan, Drs. G. H. Tovey and F. W. Lewis, Dr. J. Apley, Mr. Anthony Palin, Professor Milnes Walker, Mr. R. Belsey, Dr. S. Curwen, Mr. W. A. Jackman, Mr. R. V. Cooke, Mr. R. V. Cooke and Professor C. B. Perry, Dr. Robert Warin, Mr. A. L. Eyre-Brook, Mr. G. L. Alexander and Mr. D. G. Phillips.

The Third Meeting was held on December 14th, when Drs. Smart and Apley gave an account of their impressions of "**Medicine in America**". This was illustrated by a remarkable series of colour photographs, showing well-known cities, hospitals and physicians.

### British Orthopaedic Association

The British Orthopaedic Association held their three-day Annual Meeting in Bristol on October 13th to 15th. The last time they were in Bristol was in 1927. The first morning's meeting was confined to the Fellows and was largely a business meeting. The afternoon's meeting included a joint paper by Dr. A. L. Taylor and Dr. P. H. Beamish on "**Radiology and Histopathology of Malignant Bone Tumours**". This was followed by a joint paper by Dr. R. C. Tudway and Dr. S.

Curwen on the **" Radiotherapy of Bone Tumours ".** [This paper was to have been given by the late Dr. Raban.]

The Friday morning meeting was devoted to a discussion on Lumbar Intervertebral Disc Protusions. Friday afternoon was devoted to a clinical meeting held at Winford Orthopaedic Hospital, where cases were shown by all the members of the staff and also by Professor Milnes Walker (by invitation). Saturday morning was devoted to further papers, including one by Mr. G. L. Alexander on **" Protrusion of Cervical Discs ".**

### Devon and Exeter Medico-Chirurgical Society

At a clinico-pathological meeting on November 17th, 1949, Dr. C. P. Warren showed a post-mortem specimen of congenital pyloric stenosis occurring in a six-weeks old pedigree Scottish terrier puppy, and subsequently described two cases of cardiac catastrophe the onset of which had simulated abdominal surgical emergencies.

Dr. G. Stewart Smith demonstrated and contrasted, by means of lantern slides, two of the rarer types of thyroid enlargement, Hashimoto's and Riedel's.

Dr. Stewart Smith further demonstrated two unusual cord lesions: one of tuberculoma involving the third and forth cervical segments which must have been present for at least twenty months before pulmonary lesions were radiologically visible; and one of necrosis of cord following X-ray treatment of a supposed case of carcinoma of the middle ear.

At a meeting on December 1st, 1949, Dr. F. O. MacCullum spoke of **" The Role of the General Practitioner in Epidemiological Research in Virus Diseases ".**

Virus disease, he said, is coming out of the laboratory into the field. By taking accurate notes of even a few cases the G.P. could add to the sum of knowledge in this direction as had already been done by Dr. Pickles of Wensleydale. Incubation periods could be verified by noting single and only possible contacts. Material for the diagnosis of virus diseases is readily obtainable by the G.P. Such material is wanted for laboratory investigation in cases of suspected influenza. Where does influenza go in the summer? Where does an influenza epidemic start? Vaccines prepared and given in advance may confer immunity to influenza but it must be confessed that these were often ineffective owing to a change of strain in the virus.

Atypical pneumonias, characterized by acute onset, intense headache, malaise, depression, hacking cough and absence of fever or other physical signs were probably virus infections. One of these—so called Q Fever, due to rickettsia Burneti—appeared, at any rate in a proportion of cases, to be milk-borne. The incubation period was fourteen to twenty-five days and rashes were rare. The rickettsia could be excreted symptomlessly by cows, sheep and goats over a period as long as three months and had been found, though rarely, in cattle ticks and dog ticks. No serological tests were as yet available in this country for differentiating strains of the virus of polio-myelitis and the viruses responsible for a great group of cases labelled encephalitis had still to be worked out. The syndrome of lymphocytic meningitis was probably caused by many different viruses.

In eighty of a hundred cases investigated the virus had not been identified. The distribution of cowpox in this country had not yet been fully mapped. Dr. MacCullum asked for scrapings from suspected cowpox lesions.

At a meeting on December 15th, 1949, Dr. G. Stewart Smith discussed the fate of the over-sixty-fives. A summary of his lecture has been published in the *Lancet.*

### South Western Laryngological Association

A meeting of this Association was held at the Salisbury Combined Hospitals on October 3rd, 1949, Mr. W. H. Bradbeer in the chair. Mr. C. A. Hutchinson demonstrated the technique of the fenestration operation. In the afternoon, at the Salisbury Infirmary he showed several cases of fenestration ; two cases of malignant disease of the larynx, a thyroid hondroma, and cases of deafness associated with Paget's disease and with osteogenesis imperfecta.

The first meeting of 1950 was held at the Bristol General Hospital on January 7th. The following cases were shewn and discussed:

Mr. E. Watson-Williams : cyst of the nasal vestibule; severe but symptomless atrophic rhinitis; keratosis pharyngis.

Mr. G. R. Scarff: " nature's radical mastoid " ; pemphigus of the larynx; tuberculoma of the larynx.

Mr. J. Angell James: carcinoma of the ethmoid; carcinoma of the larynx; rhinolith.

Mr. H. D. Fairman: conductive deafness; Menière's disease; chronic pharyngitis.

The next meeting will be held at Truro on April 1st.

### The West of England and Wales Society of Dermatology

The inaugural meeting of the West of England and Wales Society of Dermatology was held at Bristol on Friday, October 28th, 1949. A clinical demonstration was held at the Bristol General Hospital and was followed by a discussion at which Dr. Clifford D. Evans took the chair. A list of provisional rules was drawn up and will be reviewed after one year. Membership is open to those who are particularly interested in the study of Dermatology. Three meetings will be held during each year. These will be arranged as follows:

The fourth week in October .. Bristol.
The fourth week in January .. Wales
The fourth week in April .. Gloucester, Exeter or Plymouth.

Dr. Geoffrey Hodgson and Dr. J. R. Simpson kindly offered to hold the next meetings at Cardiff (January) and Exeter (April) respectively. In each case the meeting will be on the Saturday morning and will take the form of a clinical demonstration and discussion followed by lunch. Dr. Robert Warin was elected as the co-ordinating secretary.

### Wessex Rahere Club

The Second Annual Dinner of the Wessex Rahere Club was held at the Spa Hotel, Bath on October 15th, 1949. Professor Sir James Paterson Ross, K.C.V.O., was present as Guest of Honour and some thirty-nine members attended under the chairmanship of Mr. C. E. Kindersley. The toast of " The Hospital " was linked with the name of Sir James Paterson Ross. In reply, Sir James outlined the present situation as regards reconstruction and development of Bart's and in particular the great progress which was being made at the Charterhouse site and in the provision of a residential hostel.

Membership of the Club, which is open without subscription to all Bart's men in the South-West, is now well over one hundred and increasing steadily. Professor R. A. Brocklehurst (Bristol) was unanimously elected Chairman for 1949–50 and the Hon. Secretary re-elected. The Third Annual Dinner will be in Bristol on October 21st, 1950. There will also be a dinner during April, 1950, at Taunton. It is hoped that any Bart's men who are not yet in touch with the Club, will apply to the Hon. Secretary (Mr. A. Daunt Bateman, 3 Circus, Bath) so that they can be kept informed of future meetings.

### British Medical Association

### Bath, Bristol and Somerset Branch

*Officers*, 1949–50. *President*, Dr. Percy Phillips. *President-elect*, Dr. W. J. Petty. *Vice-Presidents*, Dr. R. G. Gordon and Mr. J. R. Nicholson-Lailey. *Honorary Secretary*, Dr. O. E. L. Sampson. *Honorary Treasurer*, Dr. C. H. G. Price.

*Programme for* 1950. February 22nd—Bath. March 9th—Taunton. April 26th—Bristol (A Clinical Meeting). May 24th—Weston-super-Mare.

### Gloucestershire Branch

December 8th. Mr. H. Mower, **" Some everyday Ear, Nose and Throat Conditions ".**

January 12th. Mr. G. C. Dorling, F.R.C.S., **" Indications for Surgery in Gastric and Duodenal Ulcers ".**

February 9th. Dr. E. N. Davey, **" Blood Transfusion to-day ".**

March 9th. Dr. Donald Teare, **" Medical Methods in Criminology ".**

April 13th. Clinical Meeting.

May 11th. Annual Meeting (business).

June 8th. (Stroud) President's Guest.

### Bristol Division

Dr. Charles Hill addressed the Division on Wednesday, November 23rd, at the Royal Fort, Bristol University. His subject was **" The B.M.A. and all that "**. Members were allowed to introduce visitors and about half the audience of over 300 persons appeared to be non-medical. Dr. Hill gave a brief account of the organization of the Association, emphasizing that its main function was scientific, though it was apt to be known to the public only by the small part of its work which was devoted to medico-political matters.

He described the extent of its services, i.e. the work of the Central organization, its library, its overseas branches and relations with affiliated associations in the Dominions, its Public Relations Office, its Educational Department including medical films and the special Journals which it issued in addition to the *B.M.J.* He announced that it was about to produce a popular Health Journal and reminded members that the subscription is now four guineas. He then gave a brief account of the World Medical Association, of which he is President, and its relations with W.H.O. (a " Government " body) and concluded with a few tips for broadcasters from his personal experience.

On January 25th, 1950, in the University, Dr. J. A. Gorsky delivered a B.M.A. lecture on **" The Medico-Legal Investigation in Some Murder Cases "**.

### West Somerset Division

This Division held a Clinical Meeting at Yeovil District Hospital on November 24th, 1949, when Mr. E. G. Tuckwell, F.R.C.S., gave a most interesting talk on **" Sympathectomy "**.

---

## Figures and Tables

**Figure f1:**